# Impact of hippocampectomy on seizure freedom in temporal encephaloceles: A systematic review and individual participant data meta‐analysis

**DOI:** 10.1002/epi4.70036

**Published:** 2025-04-10

**Authors:** Panagiota‐Eleni Tsalouchidou, Alexandros Matsingos, Wiebke Hahn, Katja Menzler, Susanne Knake

**Affiliations:** ^1^ Epilepsy Center Hessen, Department of Neurology Philipps University Marburg Marburg Germany; ^2^ Second Department of Neurology, Attikon University Hospital National and Kapodistrian University of Athens Athens Greece; ^3^ Department of Psychiatry and Psychotherapy Philipps University Marburg Marburg Germany; ^4^ LOEWE‐Research‐Cluster for Advanced Medical Physics in Imaging and Therapy (ADMIT) TH Mittelhessen University of Applied Sciences Giessen Germany

**Keywords:** epilepsy surgery, hippocampectomy, seizure freedom, temporal encephaloceles, temporal lobe epilepsy

## Abstract

**Objective:**

Temporal encephaloceles (TEs) are increasingly recognized as a cause of MRI‐negative temporal lobe epilepsy (TLE). The optimal surgical approach for TE‐related refractory epilepsy remains unclear, particularly regarding the necessity of excluding mesiotemporal structures such as the hippocampus, which may lead to worse neuropsychological outcomes. This study evaluates the impact of hippocampectomy on achieving seizure freedom in patients with TE‐related epilepsy through a systematic review and individual participant data (IPD) meta‐analysis.

**Methods:**

A systematic literature review was conducted across Medline, Google Scholar, Embase, and Web of Science, identifying studies reporting surgical outcomes in TE‐related epilepsy. Studies were included if they provided at least 12 months of follow‐up and reported seizure outcomes using Engel or ILAE classification. The primary outcome was postsurgical seizure freedom (Engel Class IA or ILAE Class 1). A mixed‐effects logistic regression model was used to compare outcomes between patients who underwent hippocampectomy and those who did not. Heterogeneity was assessed using *τ* ^2^ and *I* ^2^ statistics.

**Results:**

The meta‐analysis included 23 studies with a total of 155 surgically treated patients. The primary analysis did not identify a statistically significant difference in seizure freedom between patients who underwent hippocampectomy and those who did not (Risk Ratio [RR] = 0.66, 95% Confidence Interval [CI]: 0.29–1.52, *p* = 0.329). Other covariates, including sex, duration of epilepsy, presence of additional epileptogenic lesions, and the use of invasive presurgical evaluation, were not significant predictors of seizure freedom. The *I* ^2^ statistic indicated moderate heterogeneity (54.68%).

**Significance:**

This IPD meta‐analysis suggests that hippocompectomy does not significantly impact seizure freedom in patients with TE‐related epilepsy and should not be part of a universal approach when determining the optimal surgical strategy. These results reinforce the need for an individualized approach, considering patient‐specific factors to optimize surgical decision‐making in TE‐related epilepsy.

**Plain Language Summary:**

Temporal encephaloceles (TEs) can cause drug‐resistant epilepsy, often requiring surgical management for seizure control. Given the variety of surgical techniques available, the optimal approach remains uncertain, particularly regarding the necessity of hippocampectomy, which may impact neuropsychological outcomes. This one‐stage individual participant data meta‐analysis found no significant difference in seizure freedom between patients who underwent hippocampectomy and those who did not. These findings suggest that hippocampectomy should not be routinely performed and highlight the importance of individualized surgical decision‐making for patients with TEs.

**Key points**

- Hippocampectomy does not significantly impact seizure freedom in TE‐related epilepsy (RR = 0.66, *p* = 0.329).
- Sex, disease duration, invasive presurgical evaluation, and additional epileptogenic lesions were not significant predictors of outcome.
- Findings support an individualized surgical approach, avoiding routine hippocampectomy when mesiotemporal involvement is uncertain.

## 1 INTRODUCTION

Temporal encephaloceles (TEs) are protrusions of brain parenchyma through defects of the meninges and skull base in the temporal region.^1^ They can cause various neurological symptoms, including epilepsy, and are considered one of the hidden causes of MRI‐negative temporal lobe epilepsy (TLE). The primary challenge lies in their identification, given their small size, the limited awareness, and the constraints of MRI in detecting minor bone deficits.^2^, ^3^

Once TEs are identified, a subsequent challenge arises in determining their potential role as an epileptogenic lesion in patients with TLE. The literature provides evidence of numerous patients with TEs achieving postsurgical seizure freedom,^4^, ^5^, ^6^, ^7^, ^8^ with TEs identified in up to 12.5% of patients with MRI‐negative drug‐resistant epilepsy. Notably, TEs are frequently identified in healthy individuals^3^, ^9^ or patients with other conditions, such as isolated cerebrospinal fluid (CSF) leaks.^10^ Multiple case series have shown a common occurrence of increased intracranial hypertension and elevated body mass index (BMI) in patients with TEs.^11^, ^12^ Moreover, TEs are associated with other epileptogenic lesions,^13^, ^14^, ^15^ often presenting bilateral,^16^ and are also found in patients with extratemporal epilepsy.^3^ Consequently, the presurgical evaluation of patients with TE‐related drug‐resistant epilepsy can be challenging.

A further critical challenge in this context is the lack of consensus on the optimal surgical approach for TE‐related epilepsy, with studies offering various recommendations regarding the surgical management of TEs. The literature presents a range of techniques, from lesionectomy to anterior temporal lobectomy, with or without hippocampectomy.^17^ Some case series suggest that more extensive methods, such as standard temporal lobectomy, may result in more favorable postsurgical outcomes.^18^ In contrast, other studies present postsurgical seizure freedom with more restrictive techniques, such as lesionectomy.^4^, ^7^, ^8^, ^19^, ^20^

A critical issue in TE‐related epilepsy surgery is determining the extent of mesiotemporal involvement and the necessity of hippocampal resection to achieve better postsurgical outcomes.^19^, ^21^ The decision to include hippocampal resection is crucial, as it carries potential risks of neuropsychological decline.^22^, ^23^, ^24^ This uncertainty complicates the choice of the best surgical strategy to treat patients with TE‐related epilepsy.

Therefore, we aimed to examine the impact of hippocampal resection on achieving seizure freedom in patients with TE‐related epilepsy. Given the limited number of small case series available in the literature, we conducted a systematic review and individual participant data (IPD) meta‐analysis to compare postsurgical outcomes between patients who underwent TE surgery with hippocampectomy and those without.

## 2 MATERIALS AND METHODS

### 2.1 Literature search strategy and search terms

A systematic literature review across four major databases: Medline, Google Scholar, Embase, and Web of Science was performed to identify studies reporting outcomes of surgically treated patients with epilepsy and TEs. The search strategy included the following terms: temporal encephaloceles or temporal meningoencephalocele, epilepsy, and epilepsy surgery. The literature search was last updated on January 31, 2025, capturing all studies published until that date. A detailed overview of the search strategy is provided in the supplement (Table S1). The study was conducted following a predefined protocol, registered and published in the International Prospective Register of Ongoing Systematic Reviews (PROSPERO),^25^ registration number: PROSPERO 2024 CRD42024500148. Methodology and reporting of results adhered to the Preferred Reporting Items for Systematic Reviews and Meta‐Analyses for Individual Patient Data (PRISMA‐IPD) guidelines.^26^

### 2.2 Inclusion and exclusion criteria for eligible studies

Studies were considered eligible for inclusion if they reported epilepsy patients with TEs who underwent epilepsy surgery, and the postoperative outcome was reported for at least 12 months, or according to the Engel or ILAE Classification. Given the rarity of TEs and the limited existing literature, the selected inclusion criteria encompassed both retrospective studies and case series, regardless of the level of evidence. Non‐English language articles were not included in the systematic review and meta‐analysis. In cases of duplicate patient publications from the same institutions, the most recent or most comprehensive dataset in terms of individual participant data availability was selected. To maintain consistency in comparing surgical techniques, we excluded studies that involved patients treated with Laser Interstitial Thermal Therapy (LITT), intranasal interventions, or transmastoid approaches. Additionally, studies reporting solely nonresective therapies, such as disconnection, were also excluded. Two investigators (PT and AM) independently screened the titles and abstracts of all identified citations to assess their relevance. Full texts of potentially relevant articles were subsequently obtained and reviewed by the same authors. Furthermore, reference lists of these publications were reviewed to identify any additional relevant publications.

### 2.3 Inclusion and exclusion criteria for eligible participants

Out of the included studies, patients were considered eligible for the analysis if they had a reported minimum postoperative follow‐up period of 12 months or documentation of seizure freedom according to Engel or ILAE classification. In cases where patients underwent surgery twice, the latest procedure and outcome were included in the analysis. Additionally, patients treated with LITT, endonasal, transmastoid approaches, or sole disconnection were also excluded from the meta‐analysis.

### 2.4 Data extraction and outcome definitions

This IPD meta‐analysis was conducted using individual participant data extracted directly from the published studies. No additional data were requested from the original study authors, and the analysis was based solely on the IPD available within the published reports.

Two reviewers (PT and AM) independently extracted data from each eligible study including the following: study design, year of publication, study location, total sample size, patient demographics (e.g., sex), age of epilepsy onset, age of epilepsy surgery, body mass index (BMI), additional epileptogenic lesion on MRI, type of additional epileptogenic lesion, signs of idiopathic intracranial hypertension (IIH), multiple TEs, bilateral TEs, type of invasive presurgical exploration, number of surgically treated patients, surgical method applied, side of surgery, number of surgically treated patients with and without removal of the hippocampus, postsurgical outcome, and duration of follow‐up after surgery. For variables such as BMI and sex that were reported only as summary statistics in some studies, we extracted means, standard deviations, and proportions where individual‐level data were not available. Given that the included studies were primarily retrospective case series and case reports, sequence generation was not applicable. Data checking focused on assessing data consistency and completeness across studies, as well as examining potential baseline imbalances among the included participants.

The primary outcome was seizure freedom for at least 12 months following surgery, which was prespecified as the main outcome for this review. For the meta‐analysis, outcomes were categorized into two groups: Group 1 included patients who were seizure‐free for 12 months following surgery or whose postsurgical outcomes were classified as Engel Class IA or ILAE Class 1; Group 2 included patients with less favorable outcomes, such as those classified as Engel Classes IB–ID, II–IV, ILAE Classes 2–5, or those experiencing persistent seizures beyond 12 months.

### 2.5 Risk of bias assessment

The methodological quality of the included studies was assessed by two reviewers (PT and WH) using the Joanna Briggs Institute (JBI) Critical Appraisal Checklist for case reports and case series. This tool was used to evaluate the risk of within‐study bias, focusing on potential sources related to study design, data collection, and reporting. Disagreements in assessments were resolved through discussion or consultation with a third reviewer (AM).^27^

### 2.6 Statistical analysis

#### 2.6.1 One‐stage individual participant data meta‐analysis

We performed a one‐stage individual participant data (IPD) meta‐analysis to evaluate the effect of hippocampal resection on surgical outcomes in patients with TEs. We opted for the one‐stage approach over the two‐stage approach due to the limited sample sizes of studies available on this subject in the literature.^28^ We used R version 4.4.1 (2024‐06‐14) for all analyses.^29^ A mixed‐effects logistic regression model was employed, using the surgical approach as a fixed effect, hypothesizing that the impact of hippocampectomy on seizure freedom is consistent across all studies. Additionally, a random intercept was included for each study to account for between‐study variability, acknowledging that individual studies may differ in their baseline risks due to unique study populations or methodologies.^28^ The surgical outcome was binary, defined as seizure freedom over 12 months. Missing values were imputed using mean imputation for numeric variables and mode imputation for categorical variables. The model was fitted using the glmer function from the lme4 package in R, with a binomial family for logistic regression. Maximum likelihood estimation was used to estimate the model parameters. The use of glmer enabled the appropriate modeling of the binary outcome while accounting for study‐level variability through random intercepts and estimating the fixed effects of the covariates, including the surgical approach.^30^ The primary variable of interest was the surgical approach, categorized into two groups: with hippocampectomy and without hippocampectomy. The dependent variable, surgical outcome, was binary, indicating whether the patient was seizure‐free over 12 months postsurgery. Additional covariates included sex, side of surgery, bilateral TEs, duration of the disease, invasive presurgical evaluation, and the presence of additional epileptogenic lesions. These covariates were included in the model to control for potential confounding factors that could influence surgical outcomes. Signs of IIH were not included as a covariate due to their limited reporting across studies, which would have resulted in a high proportion of missing data. The principal measure of effect for the primary outcome was the Risk Ratio (RR), calculated along with its 95% confidence intervals (CIs). The mixed‐effects logistic regression model provided estimates of the fixed effects, including the surgical approach and other covariates. As an additional analysis, we repeated the model using an alternative definition of seizure outcome, categorizing freedom from disabling seizures (Engel 1A–1D, ILAE 1–2) as the outcome measure to assess whether expanding the definition of seizure freedom would yield different results.

#### 2.6.2 Random‐effects examination and heterogeneity assessment

We accessed between‐study heterogeneity by estimating the between‐study variance (*τ* ^2^) using the rma() function in the metafor package. This estimation was performed using the Restricted Maximum Likelihood (REML) method, which accounts for variability across studies while minimizing bias in variance estimation. The *I* ^2^ statistic, which quantifies the proportion of total variation in effect estimates due to heterogeneity rather than chance, was then calculated using the formula: *I* ^2^ = 100 × (*τ* ^2^/[*τ* ^2^ + within‐study variance]).

#### 2.6.3 Sensitivity and robustness analyses

To ensure the robustness of our findings, we conducted a series of sensitivity and robustness checks. Firstly, a leave‐one‐out analysis was performed, where each study was iteratively excluded from the dataset, and the mixed‐effects logistic regression model was refitted to evaluate the impact of each study on the overall results. Risk ratios (RR) and their 95% confidence intervals (CI) were recalculated for each iteration to determine the influence of each study. Additionally, we tested the effects of different imputation methods for missing data, such as median imputation for numeric variables. We also explored alternative model specifications by excluding individual covariates, such as sex and additional epileptogenic lesions, to assess their influence on the primary outcomes.

#### 2.6.4 Subgroup and interaction effects analyses

To explore the robustness of our findings and investigate whether the effect of the surgical approach varied across different patient subgroups, we conducted subgroup analyses based on sex and disease duration. Disease duration was dichotomized using its mean value, categorizing the data into two subgroups: below and above the mean. Separate mixed‐effects logistic regression models were fitted for each subgroup, with the surgical approach as the primary variable of interest. These models included covariates such as sex (for the duration subgroup analysis), duration of the disease (for the sex subgroup analysis), invasive presurgical evaluation, and additional epileptogenic lesions while accounting for between‐study variability with a random intercept for each study. The results were reported as risk ratios (RR) with 95% confidence intervals (CI).

Furthermore, to assess whether the effect of the surgical approach interacted with other covariates, we extended the mixed‐effects logistic regression model to include interaction terms. Specifically, we examined interactions between the surgical approach and variables such as sex, disease duration, invasive presurgical evaluation, and additional epileptogenic lesions. These interaction models were fitted using the glmer function in R, presenting results as interaction estimates, standard errors, test statistics, and *p*‐values instead of risk ratios, allowing for a direct assessment of whether the surgical approach has differential effects across covariate levels.

## 3 RESULTS

### 3.1 Study selection and study characteristics

A total of 543 records were identified through database searching, with 83 from Medline, 45 from Web of Science, 269 from Embase, and 146 from Google Scholar. After removing 150 duplicate records, 393 records remained for screening. Of these, 193 records were excluded based on titles and abstracts. We sought 200 reports for full‐text retrieval and assessed their eligibility. Following a detailed review, 177 reports were excluded for the following reasons: Duplicate patient populations or studies from the same institutions (*n* = 12),^3^, ^8^, ^11^, ^16^, ^21^, ^31^, ^32^, ^33^, ^34^, ^35^, ^36^, ^37^ commentaries, letters, reviews, book chapters, conference abstracts (*n* = 41), nontemporal encephaloceles or other herniations (*n* = 4), articles not in English (*n* = 6), not explicitly reporting suboutcomes of Engel or seizure freedom (*n* = 2),^38^, ^39^ no follow‐up or follow‐up of less than 12 months (*n* = 18), asymptomatic TEs (*n* = 1), not surgically treated cases or surgical approach not mentioned (*n* = 19), cases treated with LITT or disconnection (*n* = 5), otolaryngological cases, no patients with seizures, no TEs reported (*n* = 43), and reports excluded due to endonasal resection (*n* = 3).

**FIGURE 1:**
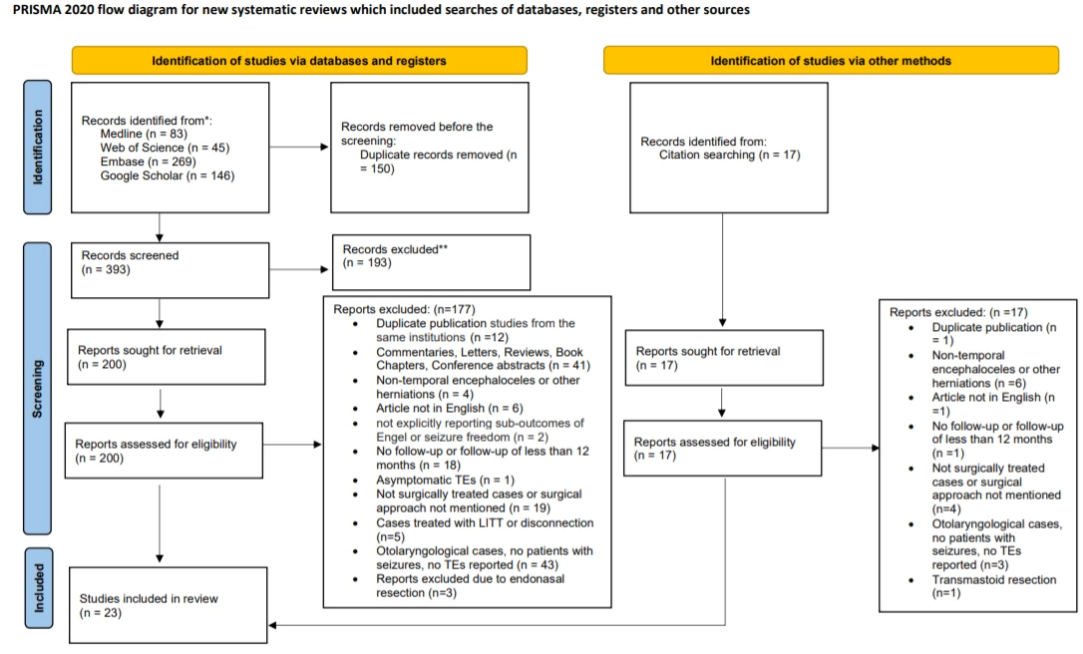
PRISMA flowchart describing the article selection process.

Additionally, 17 records were identified from citation searching. After seeking retrieval and assessing their eligibility, all 17 reports were excluded: duplicate publication (*n* = 1), nontemporal encephaloceles or other herniations (*n* = 6), articles not in English (*n* = 1), no follow‐up or follow‐up of less than 12 months (*n* = 1), not surgically treated cases or surgical approach not mentioned (*n* = 4), otolaryngological cases or patients without seizures (*n* = 3), and transmastoid resection (*n* = 1). Ultimately, 23 studies^5^, ^6^, ^7^, ^13^, ^18^, ^19^, ^20^, ^40^, ^41^, ^42^, ^43^, ^44^, ^45^, ^46^, ^47^, ^48^, ^49^, ^50^, ^51^, ^52^, ^53^, ^54^, ^55^ were included in the systematic review and the IPD meta‐analysis (Table 1). The abovementioned exclusion criteria are presented in the PRISMA diagram in Figure 1.

**TABLE 1:** Characteristics of the included studies.

Study No	Authors and Year	Study location	Nr. of surgically treated patients included	Nr. of female patients (%)	Age of epilepsy onset in years (mean ± SD, range)	Age of epilepsy surgery in years (mean ± SD, range)	BMI (mean ± SD, range)	Nr. of patients with hippocampal resection	Nr. of patients without hippocampal resection	Seizure‐free for at least 12 months after resection (%)	Follow‐up time (months) (mean ± SD, range)	Coexisting lesions on MRI (%)	Signs of IIH (%)	Bilateral TEs (%)	Multiple TEs (%)
1	Sandhu et al. 2021	USA	9	5 (56%)	32.3 (15.4, 9–60)	n/a	30.3 (7.5, 21.6–46.3)	1/9 (11%)	8/9 (89%)	0/1 (0%)	25.9 (14.3, 12–54)	1/9 (89%)	4/9 (44.5%)	0/9	3/9 (33.3%)
2	Samudraet al 2022	USA	19	12 (63%)	30.4 (43, 3–46)	40.4 (12.1, 16–61)	33.1 (8.6, 21.6–53.3)	4 (21%)	15 (79%)	3/4 (75.0%)	n/a (>12 months)^b^	0/19 (0%)	6 (31.6%)	2 (10.5%)	2 (10.5%)
3	Tsalouchidou et al. 2022	Germany	7	3 (43%)	35.57 (10.907, 20–50)	n/a	32.9 (10.4, 21.3–49.8)	4/7 (57%)	3/7 (43%)	2/4 (50%)	34.0 (20.75, 12.0–60.0)	0/7 (0%)	0/7	2/7 (28.6%)	3/7 (43%)
4	Di Giacomo et al. 2023	Italy	8	n/a^a^	n/a^a^	n/a^a^	n/a^a^	7/8 (87.5%)	1/8 (12.5%)	7/7 (100%)	n/a^a^	1/8 (13%)	n/a^a^	3/8 (37.5%)	3/8 (37.5%)
5	Bannout et al. 2018	USA	2	0 (0%)	32.5 (14.8, 22–43)	45.0 (29.7, 24–66)	n/a	0/2 (0%)	2/2 (100%)	0/0	32.5 (13.4, 23–42)	1/2 (50%)	n/a	0%	0%
6	Arslan et al. 2021	Turkey	7	2 (29%)	19.14 (5.7, 12–30)	26.86 (10.6, 20–50)	n/a	4 (57.1%)	3 (42.9%)	4/4 (100%)	50.4 (n/a, 19.2–108)	2/7 (29%)	0%	0%	0%
7	Urbach et al. 2022	Germany	14	6 (43%)	34.9 (12.8, 16–55)	n/a	32.0 (5.8, 21.3–42.7)	1/14 (7%)	13/14 (93%)	1/1 (100%)	31.3 (20.5, 12–72)	n/a	9/14 (64%)	7/14 (50%)	11/14 (78.6%)
8	Tse et al. 2020	Australia	13	8 (62%)	25 (11.9, 12–47)	37.15 (12.4, 17–58)	27.1 (8.3, 17.7–45.7)	5/13 (38%)	8/13 (62%)	3/5 (60%)	77 (56.1, 18–180)	1/13 (8%)	n/a	2 (15.4%)	3/13 (23.1%)
9	Jagtap et al. 2022	India	9	2 (22%)	13.3 (5.2, 0–17)	n/a	n/a	3/9 (33%)	6/9 (67%)	3/3 (100%)	27 (n/a, 17–44)	1/9 (89%)	n/a	0%	0%
10	Panov et al. 2016	USA	5	2 (40%)	40.4 (10.5, 23–50)	44.0 (8.1, 30–51)	n/a	4/5 (80%)	1/5 (20%)	3/4 (75%)	n/a follow‐up reported in Engel (more than 12 months)	2/5 (40%)	n/a	0%	0%
11	Buraniqi et al. 2022	USA	7	3 (43%)	11.1 (2.4, 7–14)	n/a	25.2 (5.8, 20.2–34.5)	3/7 (43%)	4/7 (57%)	2/3 (66.7%)	65.3 (32.3, 36–132)	0	n/a	0%	0%
12	Saavalainen et al. 2015	Finland	10	4 (40%)	n/a	37.6 (7.7, 22–45)	n/a	5/10 (50%)	5/10 (50%)	4/5 (80%)	34.2 (23.3, 12–74)	0	n/a	3/10 (30%)	7/10 (70%)
13	Toledano et al. 2016	Spain	5	1 (20%)	32.0 (13.9, 19–53)	47.4 (15.4, 29–62)	n/a	3/5 (60%)	2/5 (40%)	2/3 (66.7%)	range (1–4 years)	0	n/a	0%	0%
14	Giulioni et al. 2014	Italy	2	0 (0%)	31.5 (9.1, 25–38)	49.0 (11.3, 41–57)	n/a	1/2 (50%)	1/2 (50%)	1/1 (100%)	54 (8.4, 48–60)	0	n/a	0%	0%
15	Fong et al. 2019	Australia	6	2 (33.3%)	20.5 (10.3, 13–40)	29.2 (12, 16–47)	n/a	2/6 (33.3%)	4/6 (66.7%)	2/2 (100%)	14.0 (4.9, 12–24)	0	n/a	0%	0%
16	Leblanc et al. 1991	Canada	3	1 (33.3%)	22.7 (7.6, 16–31)	33.0 (6.1, 26–37)	n/a	3/3 (100%)	0/3 (0%)	3/3 (100%)	28.0 (27.7, 12–60)	0	n/a	0%	0%
17	Byrne et al. 2010	USA	3	1 (33.3%)	n/a	41.7 (15.5, 26–57)	n/a	2/3 (66.7%)	1/3 (33.3%)	1/2 (50%)	40.0 (38.6, 12–84)	0	n/a	0%	0%
18	Gasparini et al. 2018	Italy	2	0 (0%)	15.0 (4.2, 12–18)	21.0 (1.4, 20–22)	n/a	0/2	2/2 (100%)	0/0	21.0 (4.2, 18–24)	0	n/a	0%	0%
19	Swanson et al. 2021	USA	1	0	15	17	n/a	0/1	1/1 (100%)	0	12	0	n/a	0%	0%
20	Wilkins RH et al. 1993	USA	1	1	18	36	n/a	1/1	0	1/1	0	18	0	n/a	0%
21	Pejović AT et al. 2017	Serbia	2	2 (100%)	32.0 (17, 20–44)	42.0 (11.3, 34–50)	n/a	1/2 (50%)	1/2 (50%)	1/1 (100%)	12.5 (0.7, 12–13)	0	n/a	0%	0%
22	Camilo Garcia‐Gracia et al. 2024	USA	19	9 (75%)	24.7 (12.3, 3–53)	34.6 (11.8, 22–61)	n/a	2/19 (10.5%)	17/19 (89.5%)	12/19 (63.2%)	3.6 years^a^	0	n/a	2/19 (10.5%)	2/19 (10.5%)
23	Pillai R et al., 2024	India	1	1 (100%)	19	24	n/a	0	1 (100%)	1/1 (100%)	15 months	0	n/a	0%	0%

Abbreviations: BMI, Body Mass Index (kg/m^2^); IIH, Idiopathic Intracranial Hypertension; n/a, Data not available for that study or category; TE, Temporal Encephaloceles.

^a^ Summary statistics available sum statistics available for the total population, not for the included population.

^b^ Summary statistics available as median.

### 3.2 Selection, characteristics, and demographics of eligible patients

Of the included studies, a total of 167 patients were initially considered for eligibility for the IPD meta‐analysis. Of these, seven patients (4.2%) were excluded due to a follow‐up period of <12 months, one patient (0.6%) was excluded due to nonresective surgical treatment involving disconnection, three patients (1.8%) were excluded due to treatment with Laser Interstitial Thermal Therapy (LITT), and one patient (0.6%) was excluded due to not undergoing surgical treatment. Finally, a total of 155 surgically treated patients with TEs were included in the final IPD meta‐analysis. During the process of checking the IPD, no significant issues were identified, and all data related to surgical approach and outcomes were available, consistent, and accessible for all included patients across the studies. Certain variables, such as BMI and sex, were only reported as summary statistics in some of the included studies. For these variables, means, standard deviations, and proportions were extracted and are presented in Table 1. The detailed number and characteristics of the included patients from each study are presented in Table 1, while the baseline characteristics are presented in Table 2.

**TABLE 2:** Baseline characteristics of the included participants.

Surgical approach (*n* = 155)	
With hippocampectomy	56 (36.1%)
Without hippocampectomy	99 (63.9%)
Postsurgical outcome (*n* = 155)	
Seizure‐free over 12 months (Engel IA or ILAE 1)	112 (72.3%)
Not seizure‐free over 12 months	43 (27.7%)
Duration of follow‐up after surgery (months) (*n* = 107)	25.0 ± 34.57 (12–180)
Sex (*n* = 136)	Female: 63 (46.3%)
Age of epilepsy onset (years) (*n* = 155)	23.42 ± 15.20 (0–60)
Age of epilepsy surgery (years) (*n* = 101)	36.83 ± 12.53 (16–66)
Duration of the disease until surgery (years) (*n* = 89)	9.72 years (mean) ± 7.9 (0–36)
Side (*n* = 154)	
Left	96 (61.9%)
Right	58 (37.4%)
BMI (kg/m^2^)	29.18 ± 8.15 (16.40–53.30)
Additional epileptogenic lesion on MRI	9 (5.8%)
Type of lesion	
Hippocampal sclerosis	*n* = 7
Tumor in the mesial temporal lobe	*n* = 1
Amygdala enlargement	*n* = 1
Invasive presurgical evaluation (*n* = 27)	
Stereoelectroencephalography (SEEG)	*n* = 21
Electrocorticography (ECoG)	*n* = 5
Grid electrode	*n* = 1

### 3.3 Risk of bias assessment results

The risk of bias assessment for the included studies, conducted using the JBI Critical Appraisal Checklist for case series and case reports, revealed moderate methodological quality across the studies. Most studies met key criteria such as clear inclusion criteria, reliable measurement methods, and appropriate statistical analysis. However, some studies lacked consecutive inclusion of participants and had incomplete reporting of demographic data, leading to potential biases. A few studies were noted for reporting limited data. The detailed risk of bias assessment for each study is provided in Supporting Information for further reference Table S2.

### 3.4 Statistical analysis

#### 3.4.1 One‐stage individual participant data meta‐analysis

This individual participant data (IPD) meta‐analysis evaluated the role of hippocampectomy in surgical outcomes for patients with temporal encephaloceles (TEs) and temporal lobe epilepsy (TLE) by analyzing 155 surgically treated patients from 23 studies. The primary analysis did not identify a statistically significant difference in seizure freedom between patients who underwent hippocampectomy and those who did not (Risk Ratio [RR] = 0.66, 95% Confidence Interval [CI] = 0.29–1.52, *p* = 0.329). While the confidence interval suggests variability in potential outcomes, these findings indicate that hippocampectomy was not a significant predictor of achieving seizure freedom in TE‐related epilepsy. Importantly, the baseline probability of seizure freedom was high, as reflected by a statistically significant intercept in the mixed‐effects logistic regression model (β = 2.64, SE = 0.88, *p* = 0.0029, OR = 13.95, 95% CI: 2.46–78.99). This suggests that a substantial proportion of patients with TE‐related epilepsy benefit from surgery, regardless of the specific approach. Other covariates, including sex, side of surgery, bilateral TEs, duration of the disease, invasive presurgical evaluation, and additional epileptogenic lesions, were not significant predictors of the postsurgical outcome as well.

As an additional analysis, we repeated the model using an alternative definition of seizure outcome, categorizing freedom from disabling seizures (Engel 1A–1D, ILAE 1–2) as the outcome measure to assess whether expanding the definition of seizure freedom would yield different results. This analysis did not show a statistically significant effect of hippocampectomy on seizure outcomes, consistent with the primary findings. The results of this additional analysis are presented in Supporting Information as 2.4 Additional Analysis (Engel 1A–1D or ILAE 1–2), Table S10.

**FIGURE 2:**
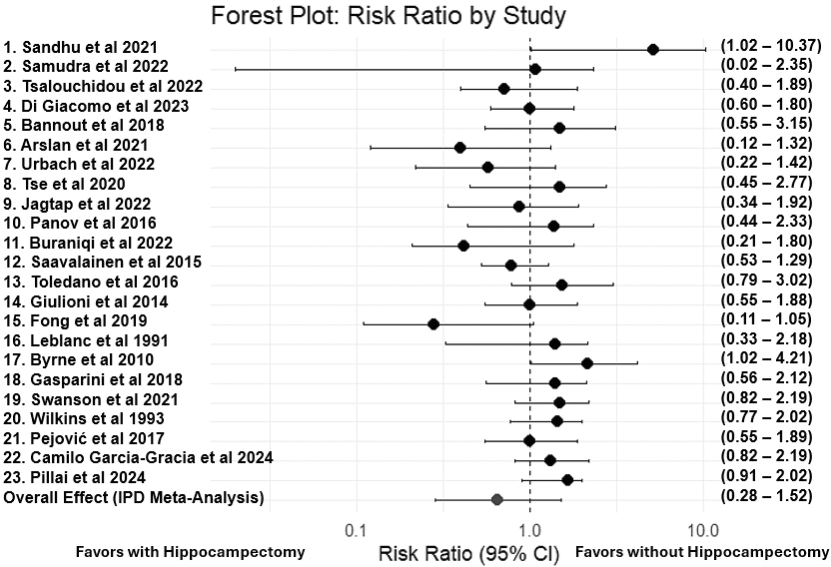
Forest Plot of Risk Ratios Comparing Seizure‐Free Outcomes Between Surgical Approaches With and Without Hippocampectomy: Each study is represented by a square, with horizontal lines indicating the 95% confidence intervals. The overall pooled estimate from the one‐stage IPD meta‐analysis suggests that hippocampectomy does not significantly increase the likelihood of achieving seizure freedom compared to approaches without hippocampectomy, as indicated by a Risk Ratio (RR) of 0.66 (95% CI: 0.28–1.52).

The results of the primary analysis are presented in Table 3, which provides the fixed effects, corresponding risk ratios, and 95% confidence intervals. Figure 2 shows the forest plot depicting the overall effect of the surgical approach on seizure freedom. Study‐specific risk ratios and their confidence intervals were calculated using log transformations and Bayesian estimates for single‐patient studies. The overall pooled effect from the one‐stage IPD meta‐analysis is displayed at the bottom of the plot, reflecting the estimated risk ratio after adjusting for all included studies and covariates.

**TABLE 3:** Summary of mixed‐effects logistic regression model results.

Effect	Term	Estimate	SE	Statistic	*p*‐Value	Risk ratio	CI lower	CI upper
Fixed	(Intercept)	2.635	0.884	2.979	0.003	13.952	2.464	78.996
Fixed	Surgical Approach without hippocampectomy	−0.417	0.427	−0.977	0.329	0.659	0.285	1.522
Fixed	Sex	−0.451	0.297	−1.520	0.128	0.637	0.356	1.139
Fixed	Side right	−0.071	0.403	−0.177	0.860	0.931	0.422	2.051
Fixed	TEs bilaterally	−0.141	0.538	−0.263	0.793	0.868	0.302	2.493
Fixed	Duration of the disease	−0.031	0.031	−1.018	0.309	0.969	0.910	1.0299
Fixed	Invasive presurgical evaluation	0.013	0.483	0.028	0.978	1.013	0.392	2.614
Fixed	Additional epileptogenic lesion	0.940	1.117	0.842	0.400	2.562	0.286	22.898

#### 3.4.2 Random‐effects examination and heterogeneity assessment

The random‐effects analysis revealed that the variance of the random intercepts for the included studies was 0.02 (*τ* ^2^ = 0.020). The within‐study variance was approximately 0.017. The *I* ^2^ statistic was calculated to be 54.68%, indicating moderate heterogeneity. Detailed results of this analysis are presented in Table S3.

#### 3.4.3 Sensitivity and robustness analyses

The sensitivity analyses confirmed the robustness of the findings. When excluding each study one at a time, the risk ratios for the surgical approach without hippocampectomy ranged from 0.29 to 1.09, with no significant deviations in the overall trend (Table S4). Median imputation for missing data yielded a similar risk ratio (RR = 0.69, 95% CI = 0.29–1.60, *p* = 0.383) (Table S5). Additionally, testing different model specifications, such as excluding the covariates sex (RR = 0.71, 95% CI = 0.17–2.90, *p* = 0.635) and additional epileptogenic lesions (RR = 0.55, 95% CI = 0.13–2.27, *p* = 0.408), did not significantly alter the primary findings (Table S6). These consistent results across various sensitivity analyses underscore the reliability of the primary outcome. Detailed results of these analyses are presented in Tables S4–S6.

### 3.5 Subgroup and interaction effects analyses

Subgroup analyses were conducted to explore whether the effect of the surgical approach varied across different patient characteristics, such as sex and disease duration. For males, the risk ratio (RR) for achieving seizure freedom with surgery without hippocampectomy was 1.57 (95% CI: 0.22–11.37), and for females, it was 0.28 (95% CI: 0.03–2.87). Similarly, when analyzing disease duration, patients with a duration ≤9.72 years (mean) had an RR of 0.34 (95% CI: 0.03–3.87), while those with a longer duration had an RR of 1.16 (95% CI: 0.21–6.42) (Table S8). None of these subgroup analyses reached statistical significance, as all confidence intervals included 1.0, suggesting no strong evidence that the effect of the surgical approach differs significantly by sex or disease duration. Detailed results are provided in Tables S7 and S8.

Finally, the interaction effects analysis did not reveal any statistically significant interactions between the surgical approach and other covariates, indicating that the relationship between the surgical approach and outcomes is consistent across different levels of the covariates. Detailed results are provided in Table S9.

## 4 DISCUSSION

The surgical management of TEs presents significant challenges, particularly in deciding the best surgical approach to achieve seizure freedom. Current literature provides inconclusive findings regarding the optimal surgical strategy for patients with TE‐related epilepsy, with reported procedures varying widely, including lesionectomy and anterior temporal lobectomy, both with and without resection of the mesiotemporal structures, and all reporting favorable postsurgical outcomes in achieving seizure freedom.^4^, ^56^, ^57^ Our findings are in line with the existing literature, demonstrating that favorable seizure outcomes can be achieved across different resection strategies and highlighting the overall benefit of surgical treatment for patients with TEs.

Both Zhou et al. (2024) and Khoudari et al. (2024) examined the surgical management of TE‐related epilepsy, with Zhou et al. providing a systematic review and data synthesis of various surgical techniques, while Khoudari et al. compared anterior temporal lobectomy (ATL) to more limited approaches. The authors acknowledge that the heterogeneity in surgical strategies complicates the distinction between limited and extensive resections, particularly in cases involving resection of the temporal pole and hippocampus. Although some procedures are categorized as limited compared to ATL, they often still involve hippocampal removal, which plays a crucial role in neuropsychological outcomes and may increase the risk of cognitive impairment.

To address this issue, this one‐stage IPD meta‐analysis specifically examined the impact of hippocampal resection, avoiding comparisons among broader surgical categories by focusing on whether hippocampectomy influences seizure freedom. The findings indicate that hippocampal removal was not a significant predictor of seizure freedom in surgically treated patients with TEs, suggesting that it should not be routinely performed in all patients with TEs and should be considered for carefully selected cases based on individualized evaluation, such as in patients with mesial temporal lesions like hippocampal sclerosis or tumors.

The IPD meta‐analysis offers several advantages, particularly in its ability to account for patient‐level variability and provide a more precise assessment of individual factors, such as coexisting epileptogenic lesions, that influence surgical outcomes. In this analysis, none of the examined covariates—including sex, disease duration, additional epileptogenic lesions, invasive presurgical evaluation, bilateral TEs, or surgical side—were found to significantly influence postsurgical seizure freedom. This suggests that these factors do not confound the relationship between the surgical approach and seizure outcomes in TE‐related epilepsy. Subgroup analyses revealed no significant differences in outcomes based on patient characteristics like sex or disease duration, with risk ratios for achieving seizure freedom with surgery without hippocampectomy showing no clear variation between these groups.

Unlike previous meta‐analyses,^57^ we did not find an association between invasive presurgical evaluation and worse postsurgical outcomes. This discrepancy may stem from differences in meta‐analysis methodologies and the small sample sizes in the existing literature. Additionally, poorer outcomes in patients undergoing invasive evaluations may reflect an inherent selection bias, as these procedures are typically reserved for more complex epilepsy cases. In such instances, the severity of epilepsy—rather than the evaluation itself—may be the primary factor influencing surgical outcomes.

Finally, the additional analysis using an alternative definition of seizure outcome, categorizing freedom from disabling seizures (Engel 1A–1D, ILAE 1–2) as the outcome measure, did not yield significantly different results, further reinforcing our primary conclusion that hippocampectomy does not necessarily impact seizure freedom in patients undergoing surgery for TE‐related epilepsy.

### 4.1 Limitations

This IPD meta‐analysis has some limitations. First, the existing literature and, consequently, the included studies primarily consist of case series and case reports, which may introduce variability and affect the generalizability of the findings due to their retrospective nature and inherent publication bias favoring positive surgical outcomes. Additionally, the heterogeneity among the included studies, as indicated by the moderate *I* ^2^ value, suggests that variability in outcomes could be attributed to differences between studies, including variations in patient selection criteria and surgical techniques. Finally, the absence of documented postsurgical neuropsychological outcomes in most of the included studies limits the ability to comprehensively assess the impact of more extensive surgical techniques, including hippocampal resections, on neuropsychological outcomes when determining the most appropriate surgical approach for patients with TEs.

## 5 CONCLUSIONS

In conclusion, this IPD meta‐analysis suggests that hippocampectomy is not required in all cases of TE‐related epilepsy to achieve seizure freedom. Instead, a personalized surgical approach should be prioritized, carefully balancing the potential benefits of hippocampal resection against its risks, particularly cognitive impairment, which was not fully assessed in the included studies. Tailoring surgical decisions to individual patient characteristics, such as the epileptogenic network, coexisting mesial temporal lesions, and neuropsychological assessment, may optimize postsurgical outcomes while minimizing unnecessary hippocampal resections. Future research should focus on refining patient selection criteria for hippocampectomy and investigating its long‐term cognitive effects to ensure the safest and most effective treatment strategies for patients with TE‐related epilepsy.

## FUNDING INFORMATION

None.

## CONFLICT OF INTEREST STATEMENT

All authors have no conflicts of interest to disclose with respect to this study.

## ETHICAL PUBLICATION STATEMENT

We confirm that we have read the Journal's position on issues involved in ethical publication and affirm that this report is consistent with those guidelines.

## Supporting information


Tables S1‐S10.

## Data Availability

The data that support the findings of this study are available from the corresponding author upon reasonable request.
