# Supplementary material for: Impact of hippocampectomy on seizure freedom in temporal encephaloceles: A systematic review and individual participant data meta‐analysis
Source: Epilepsia Open. 2025 Apr 10;10(3):796–808. doi: 10.1002/epi4.70036 (PMC12163523; doi:10.1002/epi4.70036)
Supplement: Supplementary file 1 — Tables S1‐S10. [file EPI4-10-796-s001.docx]

**Supplementary Material:**

- 1. **Table S1: Search Strategy Table**

| **Database** | **Search Strategy** |
| --- | --- |
| Google Scholar | epilepsy" AND "temporal encephaloceles" OR "temporal meningoencephalocele" AND "epilepsy surgery" |
| PubMed/ Medline | (((epilepsy) AND (temporal encephaloceles)) OR (temporal meningoencephaloceles)) AND (epilepsy surgery) |
| Embase | ('epilepsy'/exp OR epilepsy) AND ('epilepsy surgery'/exp OR 'epilepsy surgery' OR (('epilepsy'/exp OR epilepsy) AND ('surgery'/exp OR surgery))) AND ('temporal encephaloceles' OR (temporal AND ('encephaloceles'/exp OR encephaloceles))) OR 'temporal meningoencephalocele' OR (temporal AND ('meningoencephalocele'/exp OR meningoencephalocele)) |
| Web of Science | (((ALL=(epilepsy)) AND ALL=(temporal  encephaloceles)) OR ALL=(meningoencephaloceles)) AND ALL=(epilepsy surgery) |

1. **Table S2: Risk of Bias Assessment**

| **Study** | **Clear Criteria for Inclusion** | **Condition Measured Reliably** | **Valid Identification Methods** | **Consecutive Inclusion** | **Complete Inclusion** | **Clear Reporting of Demographics** | **Clear Reporting of Clinical Information** | **Clear Outcome Reporting** | **Clear Reporting of Site Information** | **Appropriate Statistical Analysis** | **Overall Appraisal** |
| --- | --- | --- | --- | --- | --- | --- | --- | --- | --- | --- | --- |
| Sandhu et al., 2024 | Yes | Yes | Yes | Yes | Yes | Yes | Yes | Yes | Yes | Yes | Include |
| Samudra et al., 2023 | Yes | Yes | Yes | Yes | Yes | Yes | Yes | Yes | Yes | Yes | Include |
| Tsalouchidou et al., 2022 | Yes | Yes | Yes | Yes | Yes | No | Yes | Yes | Yes | Yes | Include |
| Di Giacomo et al., 2023 | Yes | Yes | Yes | Yes | Yes | Yes | Yes | Yes | Yes | Yes | Include |
| Bannout et al., 2018 | Yes | Yes | Yes | N/A | Yes | Yes | Yes | Yes | Yes | Yes | Include |
| Arslan et al., 2021 | Yes | Yes | Yes | Yes | Yes | Yes | Yes | Yes | Yes | Yes | Include |
| Urbach et al., 2022 | Yes | Yes | Yes | Yes | Yes | Yes | Yes | Yes | Yes | Yes | Include |
| Tse et al., 2020 | Yes | Yes | Yes | Yes | Yes | Yes | Yes | Yes | Yes | Yes | Include |
| Jagtap et al., 2022 | Yes | Yes | Yes | Yes | Yes | Yes | Yes | Yes | Yes | Yes | Include |
| Panov et al., 2015 | Yes | Yes | Yes | Yes | Yes | Yes | Yes | Yes | Yes | Yes | Include |
| Buraniqi et al., 2023 | Yes | Yes | Yes | Yes | Yes | Yes | Yes | Yes | Yes | Yes | Include |
| Saavalainen et al., 2015 | Yes | Yes | Yes | Yes | Yes | Yes | Yes | Yes | Yes | Yes | Include |
| Toledano et al., 2022 | Yes | Yes | Yes | Yes | Yes | Yes | Yes | Yes | Yes | Yes | Include |
| Giulioni et al., 2014 | Yes | Yes | Yes | No | Yes | Yes | Yes | Yes | Yes | Unclear | Include |
| Fong et al., 2019 | Yes | Yes | Yes | No | Yes | Yes | Yes | Yes | Yes | Yes | Include |
| LeBlanc et al., 1991 | Yes | Yes | Yes | Unclear | Yes | Yes | Yes | Yes | Yes | Unclear | Include |
| Byrne et al., 2010 | Yes | Yes | Yes | Unclear | Yes | Yes | Yes | Yes | Yes | Yes | Include |
| Gasparini et al., 2018 | Yes | Yes | Yes | Yes | Yes | Yes | Yes | Yes | Yes | Yes | Include |
| Swanson et al., 2021 | Yes | Yes | Yes | N/A | N/A | Yes | Yes | Yes | Yes | N/A | Include |
| Wilkins et al., 1993 | Yes | Yes | Yes | N/A | N/A | Yes | Yes | Yes | Yes | N/A | Include |
| Pejovic et al., 2017 | Yes | Yes | Yes | Unclear | Yes | Yes | Yes | Yes | Yes | Unclear | Include |
| Camilo Garcia-Gracia et al 2024 | Yes | Yes | Yes | Yes | Yes | Yes | Yes | Yes | Yes | Yes | Include |
| Pillai R et al 2024 | Yes | Yes | Yes | Yes | Yes | Yes | Yes | Yes | Yes | Yes | Include |

1. **Statistical Analysis**

**2.1. Table S3: Random Effects Examination and Heterogeneity Assessment**

| Measure | Value |
| --- | --- |
| Between-Study Variance (τ²) | 0.020 |
| Within-Study Variance (σ²) | 0.017 |
| I² (%) | 54.68% |

- 1. **Sensitivity Analyses**
     1. **Table S4: Excluding Certain Studies**

| Study ID | Risk Ratio | CI Lower | CI Upper | P value |
| --- | --- | --- | --- | --- |
| 1 | 0.70 | 0.17 | 2.90 | 0.626 |
| 2 | 0.85 | 0.15 | 4.73 | 0.849 |
| 3 | 0.70 | 0.17 | 2.90 | 0.626 |
| 4 | 0.70 | 0.17 | 2.90 | 0.626 |
| 5 | 0.65 | 0.16 | 2.74 | 0.561 |
| 6 | 1.08 | 0.23 | 5.02 | 0.918 |
| 7 | 0.70 | 0.17 | 2.90 | 0.626 |
| 8 | 0.29 | 0.05 | 1.85 | 0.192 |
| 9 | 0.70 | 0.17 | 2.90 | 0.626 |
| 10 | 0.58 | 0.13 | 2.62 | 0.476 |
| 11 | 0.70 | 0.17 | 2.90 | 0.626 |
| 12 | 0.70 | 0.17 | 2.90 | 0.626 |
| 13 | 0.81 | 0.18 | 3.62 | 0.782 |
| 14 | 0.69 | 0.17 | 2.88 | 0.612 |
| 15 | 1.09 | 0.26 | 4.61 | 0.912 |
| 16 | 0.86 | 0.20 | 3.76 | 0.840 |
| 17 | 0.73 | 0.18 | 3.05 | 0.668 |
| 18 | 0.70 | 0.17 | 2.85 | 0.616 |
| 19 | 0.68 | 0.16 | 2.80 | 0.589 |
| 20 | 0.75 | 0.18 | 3.09 | 0.692 |
| 21 | 0.69 | 0.17 | 2.90 | 0.617 |
| 22 | 0.70 | 0.17 | 2.90 | 0.626 |
| 23 | 0.70 | 0.17 | 2.90 | 0.626 |

- - 1. **Table S5: Median Imputation for Missing Data**

| Imputation Method | Risk Ratio | CI Lower | CI Upper | P value |
| --- | --- | --- | --- | --- |
| Median | 0.69 | 0.29 | 1.6 | 0.383 |

- - 1. **Table S6: Testing Different Model Specifications**

| Covariate Excluded | Risk Ratio | CI Lower | CI Upper | P value |
| --- | --- | --- | --- | --- |
| Sex | 0.71 | 0.17 | 2.90 | 0.635 |
| Additional epileptogenic lesion | 0.55 | 0.13 | 2.27 | 0.408 |

- 1. **Subgroup and Interaction Effects Analyses:**
     1. **Table S7: Subgroup Analysis by Sex**

| **Subgroup** | **Risk Ratio** | **CI Lower** | **CI Upper** |
| --- | --- | --- | --- |
| male | 1.565 | 0.216 | 11.366 |
| female | 0.277 | 0.027 | 2.866 |

- - 1. **Table S8: Subgroup Analysis by Duration of Disease**

| **Subgroup** | **Risk Ratio** | **CI Lower** | **CI Upper** |
| --- | --- | --- | --- |
| Duration ≤ 9.72 years | 0.340 | 0.030 | 3.873 |
| Duration > 9.72 years | 1.158 | 0.209 | 6.417 |

- - 1. **Table S9: Interaction Effects Analysis**

| **InteractionTerm** | **Estimate** | **Std.error** | **Statistic** | **P.value** |
| --- | --- | --- | --- | --- |
| Surgical Approach without hippocampectomy * sex | 1.548 | 1.414 | 1.094 | 0.273 |
| Surgical Approach without hippocampectomy * Duration of the disease | -0.044 | 0.076 | -0.585 | 0.557 |
| Surgical Approach without hippocampectomy * invasive presurgical evaluation | -0.408 | 1.549 | -0.263 | 0.792 |
| Surgical Approachwithout hippocampectomy * additional epileptogenic lesion | -1.533 | 1,495.648 | -0.001 | 0.999 |

- 1. **Additional Analysis**

**Table S10. Mixed-Effects Logistic Regression Results for Alternative Seizure Outcome Definition (Engel 1A–1D or ILAE 1–2)**

| Effect | Term | Estimate | Std.error | Statistic | P.value | Risk Ratio | CI Lower | CI Upper |
| --- | --- | --- | --- | --- | --- | --- | --- | --- |
| fixed | (Intercept) | -3.00 | 1.067 | -2.815 | 0.004 | 0.049 | 0.006 | 0.401 |
| fixed | Surgical Approach without hippocampectomy | 0.33 | 0.505 | 0.659 | 0.509 | 1.395 | 0.518 | 3.758 |
| fixed | sex | 0.47 | 0.354 | 1.333 | 0.183 | 1.603 | 0.800 | 3.210 |
| fixed | Side | 0.04 | 0.474 | 0.093 | 0.926 | 1.045 | 0.413 | 2.647 |
| fixed | TEs bilateral | 0.47 | 0.585 | 0.810 | 0.418 | 1.606 | 0.510 | 5.055 |
| fixed | Duration of the disease | -0.003 | 0.038 | -0.090 | 0.928 | 0.997 | 0.924 | 1.074 |
| fixed | Invasive presurgical evaluation | 0.44 | 0.514 | 0.852 | 0.393 | 1.550 | 0.566 | 4.244 |
| fixed | Additional epileptogenic lesion | -0.400 | 1.139 | -0.349 | 0.727 | 0.672 | 0.072 | 6.267 |
